# 

**Published:** 1897-02-06

**Authors:** 


### EARLY EDITION.

**Notices of Births, Deaths, and Marriages** are inserted in this column at a charge of 2s. 6d. for an announcement not exceeding four lines.

### NOTICE TO CORRESPONDENTS.

All MS., Letters, Books for Review, and other matters intended for the Editor should be addressed The Editor, "The Hospital," The Hospital Building, 28 & 29, Southampton Street, Strand, W.C.

The Editor cannot undertake to return rejected MS., even when accompanied by stamped directed envelope.

**Notice to Advertisers and Subscribers.**

All Advertisements, Orders for Copies of The Hospital, and Business Communications should be addressed to **THE MANAGER, (Not to The Editor),** The Hospital, The Hospital Building, 28 & 29, Southampton Street, Strand, London, W.C.

The Cost of Subscription, post free, is as follows:—Per week, 2½d.; per quarter, 2s. 8d.; per half-year, 5s. 3d.; per annum, 10s. 6d. Foreign:—Per annum, 15s. 2d.; payable, in all cases, in advance.

**NOTICE.—Vol. XX. of "The Hospital" (April to September, 1896), in handsome cloth binding, is now on sale at the Office of this Journal, price 7s. 6d. Cases for binding the Numbers contained in this Volume, 1s. 6d. Index to Vol. XX., 2½d., post free.**

**The Monthly Part for February is now ready, Price 6d., by post 8d.**

### The Student of Medicine.

The following questions were set at the examination held in January for the triple qualification granted by the Royal Colleges of Edinburgh and the Faculty of Physicians and Surgeons of Glasgow:—

Medicine (Three questions only to be answered).

1. Describe shortly the blood changes, and the morbid anatomy generally, in tertian ague. What symptoms arise during an acute attack?
2. Give a brief account of the morbid anatomy, symptoms, and course of bulbar paralysis (labio-glosso-laryngeal paralysis).
3. What are the usual causes of ascites? Give the physical signs of that condition.
4. Enumerate the causes of hæmaturia.

Surgery (Three questions to be answered).

1. Enumerate the usual causes of retention of urine in the male sex, in a child, an adult, and an old man respectively.
2. What are the points to be specially attended to in applying splints in a case of fracture of a long bone?
3. What are dermoid cysts, where do they occur on the head? What is their pathology? What are they apt to be mistaken for, and how should they be treated?
4. Describe an indolent or callous ulcer and describe shortly how it should be treated.

Surgical Anatomy (Both questions to be answered).

1. What symptoms are present in paralysis (*a*) of the third cranial nerve; (*b*) of the sixth cranial nerve; (*c*) of the first division of the fifth cranial nerve? Explain these symptoms anatomically.
2. What anatomical points have you to bear in mind, when clearing out the axilla, in a case of malignant disease of the glands?

Therapeutics (All the questions to be answered, including the prescription).

1. Describe the lines of treatment to be adopted in a case of cirrhosis of the liver.
2. What special lines of treatment would you follow in a case of acute rheumatism, if (1) pericarditis, or (2) hyperpyrexia, developed?
3. Give the treatment of epilepsy.

Prescription (To be written in unabbreviated Latin, with the directions for the patient in English).—Prescribe ipecacuanha for a case of acute dysentery.

Midwifery and Gynecology (Three questions to be answered, of which the last must be one).

1. Give the chief characters of the flat rickety pelvis, and describe the mechanism of labour characteristic of it.
2. You are called to a patient in the second stage of labour, with the arm of the child protruding from the vagina, the membranes having ruptured several hours previously; how would you manage such a case?
3. Mention the causes of albuminuria in pregnancy, and the dangers attendant on it. Give the symptoms and treatment.
4. (*Gynecology*).—What is pelvic haematocele? Describe the causes, symptoms, and physical signs. Give the treatment, indicating what cases call for operative interference.

Medical Jurisprudence and Hygiene. (Three questions only to be answered. The candidate may answer either No. 1 or No. 2, but must answer Nos. 3 and 4.)

1. In what forms of death would you expect the blood to be fluid or coagulated? Mention the bearing of these conditions on the post-mortem appearances.
2. What is the law relating to what is termed in medical jurisprudence the secondary causes of death? What is their bearing on the responsibility of the accused.
3. A pistol is found near a dead body. How could you determine, by chemical means, that a fortnight had elapsed since it had been fired?
4. (*Hygiene.*)—Water supply: State the respective advantages and disadvantages of a constant and of an intermittent supply of water to a civic community.

